# Understanding the Association Between Electronic Health Record Satisfaction and the Well-Being of Nurses: Survey Study

**DOI:** 10.2196/13996

**Published:** 2020-06-23

**Authors:** Saif Khairat, Lin Xi, Songzi Liu, Shilpa Shrestha, Charles Austin

**Affiliations:** 1 School of Nursing University of North Carolina at Chapel Hill Chapel Hill, NC United States; 2 Carolina Health Informatics Program University of North Carolina at Chapel Hill Chapel Hill, NC United States

**Keywords:** electronic health record, nursing, satisfaction, critical care

## Abstract

**Background:**

Intensive care unit (ICU) nurses experience high levels of burnout related to the high-stress environment. Management of electronic health records (EHR) is a contributing factor to physician burnout. However, limited research has established the relationship between the nurse’s well-being and EHR use.

**Objective:**

The objective of this study was to examine the association between EHR use and the well-being of nurses.

**Methods:**

We surveyed registered nurses employed at a major Southeastern medical center in the United States about their demographics, experience with EHRs, satisfaction with EHRs, and elements of well-being. The correlation between subgroup demographics and survey questions was examined using Kendall and Fisher tests.

**Results:**

A total of 113 ICU registered nurses responded to the survey, of which 93 (82.3%) were females. The population had a mean age of 35.18 years (SD 10.65). A significant association was found between satisfaction and well-being scores, where higher EHR satisfaction was associated with higher self-reported well-being (correlation 0.35, *P*<.001). Nurses who were unhappy with the time spent in EHR use compared with direct patient care reported higher levels of stress (*P*<.001) and isolation (*P*=.009). Older nurses reported higher dissatisfaction with the amount of time spent on EHR tasks related to direct patient care compared to younger nurses (*P*<.001).

**Conclusions:**

Although nurses reported acceptable satisfaction scores with EHR use, deeper analysis suggests that EHR indirectly affects the well-being of nurses. These findings strongly indicate that lower EHR satisfaction can impact the well-being of nurses. More research is needed to optimize the nurse-EHR experience through more user-centered design approaches.

## Introduction

### Background

The professional well-being of health care workers has been a topic of interest in research for decades, first mentioned in a study addressing physician burnout in 1981 [1]. A wealth of research has been conducted since then to analyze what burnout is, the risk factors for its development, its negative outcomes, and potential interventions to reduce its incidence. It has been defined as one having feelings of hopelessness, apathy, and the eventual inability to function effectively in one’s professional role [2,3]. In addition, burnout typically develops over time with a relatively slow onset, related to continued exposure to contributing factors [4].

Although burnout has been described in a variety of manners, three overarching themes identified by Maslach, Jackson, and Leiter [5] shape the preeminent definition of burnout: feelings of emotional exhaustion, depersonalization, and reduced personal accomplishment. Of considerable importance when discussing burnout concerning nursing is the topic of factors that contribute to its development. Factors such as high patient or unit acuity, inadequate staffing levels, and conflict with the administration were identified as key contributors to developing burnout [6]. In addition, Hunsaker et al [4] identified age as a risk factor for nurses, with younger nurses exhibiting higher levels of burnout when compared with their older colleagues. This finding is likely related to the large learning curve experienced by new nurses and can be expected to be higher in young nurses on high acuity units. Another contributing factor to the high patient or unit acuity is the relative prevalence of burnout by unit type. Research on nursing burnout has primarily focused on intensive care unit (ICU) nurses with the consensus that ICU nurses experience very high levels of burnout, related to the high-stress environment [7-9]. Longer (≥12 hours) shifts and lower education levels (below a master’s degree) were also identified as significant factors in nursing burnout development [4]. Additional studies identified nurses working in emergency departments as having elevated rates of burnout [4]. When considering other acute care settings such as medical-surgical units, burnout is less prevalent, but most notably affects younger nurses on such units [10].

The negative outcomes resulting from nurses experiencing burnout are numerous [11,12]. Of primary consideration are the mental health and general well-being of the affected nurses. Mealer et al [13] identified that approximately 21% of nurses with burnout syndrome had a concurrent diagnosis of posttraumatic stress disorder. Those nurses with both diagnoses were likely to experience high levels of anxiety and nightmares, altered perception, and attitude toward elements of their personal lives [13]. High levels of burnout also significantly increase the rates of turnover on a unit, negatively affecting nurse retention [4]. Finally, high levels of nursing burnout are correlated with decreased patient safety and satisfaction [14].

In the present technological age, a significant part of a health care provider’s day revolves around computerized charting systems, typically referred to as electronic health records (EHRs). EHRs are made up of a variety of functions such as computerized provider order entry, clinical notes, test results, clinical decision support tools (ie, drug interaction warnings and allergy warnings), electronic paging, and electronic communication with patients [15]. Although these systems have notable effects on decreasing hospital costs and increasing care quality, several issues with EHRs have been identified [16]. Nurses have indicated that the increased time spent on documentation and the abundance of checkboxes negatively impacted their nursing work [17]. Usability-specific issues related to EHR design and changes to workflow have shown to contribute to a negative perception of EHRs, and, in conjunction, contribute to decreased efficiency and satisfaction among physicians [16,18]. Decreased professional satisfaction among physicians with EHR use was reported to be because of the experience of clerical burden [15,19]. The use of EHRs and the associated clerical burden is related to increased levels of physician burnout, regardless of their satisfaction with EHRs in general [20]. In contrast, the study conducted by Harris et al [21] regarding EHR-related stress and advanced practice registered nurses (APRNs) identified negative attitudes toward EHRs as strongly associated with APRN burnout.

When considering physicians and APRNs, researchers arrive at the same conclusion: EHRs and EHR-related stress are associated with burnout and well-being [21]. These studies examined the statistical relationships between participant scores on separate measures used to identify burnout and EHR satisfaction. In this regard, these studies provide valuable information about the impact of the EHR on clinician well-being by focusing on physicians and APRNs. Like physicians and APRNs, nurses spend large quantities (up to 50%) of their work time using EHRs, although their tasks within the EHR differ [17,22]. Despite the number of studies addressing clinician well-being to EHR use, and nursing burnout and nurses’ perception of EHRs separately, a gap in the literature exists regarding the correlation between the well-being of nurses and EHR use. For these reasons, we hypothesize that EHR use negatively affects satisfaction and overall well-being for nurses.

### Objective

This study aimed to address this literature gap by examining the association of EHR use with nursing satisfaction and the overall perceived well-being of nurses in a hospital setting.

## Methods

### Setting and Participants

Participants in this study were registered nurses employed at a Southeastern medical center in the United States. The medical center has a total of over 800 beds. Participants were asked to indicate their highest degree held, which included Associate Degree in Nursing, Bachelor of Science in Nursing, Master of Science in Nursing, and Doctorate of Nursing Practice. Participants indicated the name of the unit on which they worked. These units included a variety of ICUs, stepdown units, medical-surgical floors, and others. Participating nurses were recruited via email listservs and in-person on select units. Participants completed the paper or electronic survey on a volunteer basis and were not compensated for their participation.

### Materials

The survey was developed for this study through the utilization of previously developed instruments. The demographics section asked participants about their age, gender, unit in which they worked, and the highest degree held. This section also included questions about the number of years of experience they had using the EHR and the estimated number of hours weekly that they used the EHR.

We investigated using an existing survey tool for our study; however, we could not find a survey instrument that included all measures of interest. Therefore, we built a hybrid survey on the basis of validated EHR questions from the description of technology use in practice and the Maslach Burnout Inventory. Participant satisfaction with EHR systems was measured by 3 questions (Q1-Q3) adapted from the description of technology use in practice measure developed by Shanafelt et al [15]. These questions utilized 5-point Likert scales, asking participants to rate EHR satisfaction on a scale ranging from *very dissatisfied* to *very satisfied* and *strongly disagree* to *strongly agree* for the remaining 2 questions. The well-being variable was measured by an additional 6 questions (Q4-Q9) adapted from the Maslach Burnout Inventory [5]. Each of the questions utilized a 5-point Likert scale, asking participants to indicate agreement with the provided statement by choosing *strongly disagree*, *disagree*, *neutral*, *agree*, or *strongly agree*. A copy of the paper survey is in Multimedia Appendix 1.

### Procedure

Participants were either verbally instructed or informed via an email that the survey was designed to explore any potential correlation between EHRs and the well-being of nurses. Data were collected by hard copy and electronically by Qualtrics survey, both of which were designed to complete within 5 min. Participation in the survey was voluntary, and participants could discontinue at any time. Participants who completed the paper survey were additionally given a copy of the consent form to review before completing the survey. The anonymity of these participants was maintained by not collecting the signed consent forms. Completed paper survey forms were placed in a folder for later data entry. The Qualtrics survey link was distributed electronically via email along with the consent form available for review as an attachment to the sample population. The participants were requested to contact the principal investigator or the institutional review board if they had any questions or concerns. Institutional review board approval was obtained before starting this research.

### Outcome Variable

The outcome variables were *satisfaction score* and *well-being score.* The *satisfaction* *score* measured the satisfaction with a prominent EHR system, which was the mean score of Q1 to Q3. *Well-being score* measured the degree of nursing EHR well-being experienced by an individual participant, which was the mean score of Q4 to Q9. These metrics were derived from the arithmetic mean of the accumulated score of all 9 survey questions. On a range of 0 to 5, the lower the *well-being score*, the higher the degree of burnout.

### Statistical Analysis

#### Data Analysis

Incomplete entries (either missing some demographic information or some response) were not eliminated beforehand. Instead, we performed only necessary removals before carrying out various statistical analyses. Questions 5, 7, and 8 were negative scoring questions; the responses were reversely coded for analysis. We used Pearson correlation to test the relationship between satisfaction (mean score of questions 1-3) and well-being (mean score of questions 4-9). We also used Pearson correlation to correlate each survey question with satisfaction score and well-being score.

#### Kendall Test and Fisher Test

Each subgroup’s association with every question was examined either by the Fisher test or by the Kendall test. As gender is a nominal variable, the former one was used to test its relationship with each survey question. As the remaining subgroups were ordinal, their relationship to each survey question was investigated by the Kendall test. Both tests returned *P* values, whereas the Kendall test further provided a correlation coefficient, which was between −1 and 1. The sign of this coefficient noted the positivity or negativity of the relationship, and the absolute value of it represented the relation’s strength.

## Results

### Demographic Characteristics

A total of 113 ICU registered nurses responded to the survey, of which female nurses were 82.3% (93/113) of the surveyed population, whereas males comprised 17.6% (20/113). The population had a mean age of 35.18 years (SD 10.65). Approximately 3 of 4 participants were under the age of 45 years, indicating a relatively young population. Among the participants, 87 held a Bachelor of Science in Nursing as their highest completed degree, whereas 17 participants held an Associate Degree in Nursing, 5 participants held a Master of Science in Nursing, and only 1 held a Doctorate of Nursing Practice. Table 1 displays the characteristics of the study participants.

**Table 1:** Descriptive analysis of participants population (N=113).

Characteristics		Participants, n (%)	Well-being score (SD)
Average		113 (100.0)	3.22 (0.17)
**Gender**			
	Male	20 (17.7)	3.27 (0.44)
	Female	93 (82.3)	3.23 (0.5)
**Age^a^ (years)**			
	18-24	12 (10.6)	3.29 (0.34)
	25-34	49 (43.3)	3.3 (0.38)
	35-44	22 (19.4)	3.32 (0.58)
	45-54^a^	18 (15.9)	2.89 (0.56)
	≥55	6 (5.3)	3.41 (0.63)
	N/A	6 (5.3)	—^b^
**Years of practice^a^**			
	0-1	5 (4.4)	3.6 (0.37)
	1.5-3.5^a^	34 (30.0)	3.05 (0.44)
	≥4	72 (63.7)	3.3 (0.49)
	N/A	2 (1.7)	—
**Hours per week in EHR^c^**			
	0-19	34 (30.0)	3.25 (0.52)
	20-39	59 (52.2)	3.25 (0.47)
	40+	16 (14.1)	3.15 (0.51)
	N/A	4 (3.5)	—
**Highest degree attained**			
	ADN^d^	17 (15.0)	3.12 (0.77)
	BSN^e^	87 (76.9)	3.27 (0.43)
	MSN^f^	5 (4.4)	3.09 (0.46)
	DNP^g^	1 (0.8)	2.89 (N/A^a,h^)
	N/A	3 (2.6)	—

^a^Self-reported data.

^b^Missing data

^c^EHR: electronic health record.

^d^ADN: Associate Degree in Nursing.

^e^BSN: Bachelor of Science in Nursing.

^f^MSN: Master of Science in Nursing.

^g^DNP: Doctorate of Nursing Practice.

^h^N/A: not applicable.

#### Descriptive Study of Participants

The population average of well-being score was 3.22, which was between neutral and satisfied with their experience with nursing EHR. The responses of nurses were subsequently analyzed in subgroups (Table 1). We found that nurses aged between 45 and 54 years responded to the survey questions significantly different from their peers, yielding a mean well-being score of 2.89. On the other hand, nurses who had between 1.5 and 3.5 years of experience reported a higher degree of well-being than those who practice either long or short periods. Female nurses, which contributed to 80% of the participant population, did not demonstrate a significant average well-being score comparing to that of male nurses.

### Association Between Electronic Health Record Satisfaction and Well-Being

A significant association was found between satisfaction and well-being, where a higher EHR satisfaction was associated with high self-reported nurse well-being (correlation 0.35, *P<*.001). The mean EHR satisfaction score was 3.23 (SD 0.75), and the mean well-being score was 3.22 (SD 0.48). A wider range of scores was observed in EHR satisfaction (minimum of 1, maximum of 4.7) compared with well-being score (minimum of 1.8, maximum of 4.3).

The level of satisfaction with the EHR was significantly associated with the perceived well-being of nurses, such that higher EHR satisfaction led to higher well-being scores (*P*=.002; Table 2). A significant positive relationship was found between the perceived efficiency and time spent in the EHR with overall EHR satisfaction (*P*=.001 for both).

**Table 2:** Pearson correlation coefficients of correlations between each survey question and electronic health record satisfaction and nurse well-being scores.

Question	EHR^a^ Satisfaction Items		Well-being Items	
	EHR satisfaction score	*P* value	Well-being score	*P* value
Rate level of satisfaction with EHRs	N/A^b^	N/A	0.29897	.003
EHRs have improved my efficiency	N/A	N/A	0.22655	.03
The amount of time I spend on EHR tasks related to direct patient care is reasonable.	N/A	N/A	0.33707	.007
I can manage the amount of my work well.	0.42326	<.001	N/A	N/A
After my work, I usually feel worn out and weary (reverse coding).	0.16846	.097	N/A	N/A
I can tolerate the pressure of my work very well.	0.20003	.048	N/A	N/A
One can become disconnected from this type of work (reverse coding).	0.20776	.04	N/A	N/A
I tend to think less at work and do my job almost mechanically (reverse coding).	0.28953	.004	N/A	N/A
I always find new and interesting aspects in my work.	0.01389	.89	N/A	N/A

^a^EHR: electronic health record.

^b^N/A: not applicable.

### Findings From Subgroup Analysis

We used the Kendall test and the Fisher exact test to examine whether nurses of a certain demographic group tended to respond differently to any question (Table 3). We found 3 subgroup-question pairs to be correlated. Kendall test suggested that respondents with higher ages tended to consider the amount of time they spent on EHR tasks related to direct patient care as less reasonable (*P*=.002). Besides, higher age respondents held a stronger opinion against the EHR’s positive effect on efficiency, supported by a *P* value of .03 and correlation coefficient −0.18. The last significant pair, subgroup *years of practice* versus question 4, suggested that respondents with more years of practice showed more agreement to the traceableness of their work.

**Table 3:** Kendall test for demographics and question responses.

Subgroup-question pairs	Correlation coefficient	*P* value
Age versus the amount of time spent on EHR^a^ tasks related to direct patient care is reasonable.	−0.2566	.002
Age versus EHR have improved my efficiency.	−0.1838	.03
Years of practice versus I can manage the amount of my work well.	0.2403	.007

^a^EHR: electronic health record.

### Analysis of Survey Questions

We calculated the average score of individual questions and compared them with the population average (Table 4). Among the 3 EHR-specific survey items, Question 1 to 3, participants responded most positively to *Question 1* (3.66), suggesting an overall satisfaction with the nursing EHR. However, the lower score received by *Question 3* (2.73) indicated that, on average, participants agreed that the balance between nursing EHR activities and direct patient care had not been achieved. The participants showed the highest agreement with *Question 4* and *Question 6*, which measured the non-nursing EHR-specific aspects of their work routine. In other words, the nurses were generally positive about their ability to managing workload and work-related stress. On the other hand, the lowest agreement was observed in *Question 5* and *Question 7*—participants reported feeling disconnected from the work and worn out.

**Table 4:** Mean and mode score of survey questions.

Question	Description	Mean score (SD)
1	Rate level of *satisfaction* with EHRs^a^.	3.66 (0.80)
2	EHRs have improved my *efficiency.*	3.28 (1.040)
3	Amount of *time* I spend on EHR tasks related to direct patient care is reasonable.	2.73 (0.97)
4	Usually, I can *manage the amount of my work* well.	4.00 (0.64)
5	After my work, I usually *feel worn out* and weary.	2.18 (0.88)
6	I can *tolerate the pressure* of my work very well.	3.87 (0.70)
7	Over time, one can become *disconnected* from this type of work.	2.22 (0.85)
8	Lately, I tend to think less at work and *do my job almost mechanically*.	3.35 (1.03)
9	I always find *new and interesting aspects* in my work.	3.75 (0.81)
1-9	N/A^b^	3.22 (0.49)

^a^EHR: electronic health record.

^b^N/A: not applicable.

**Figure 1:**
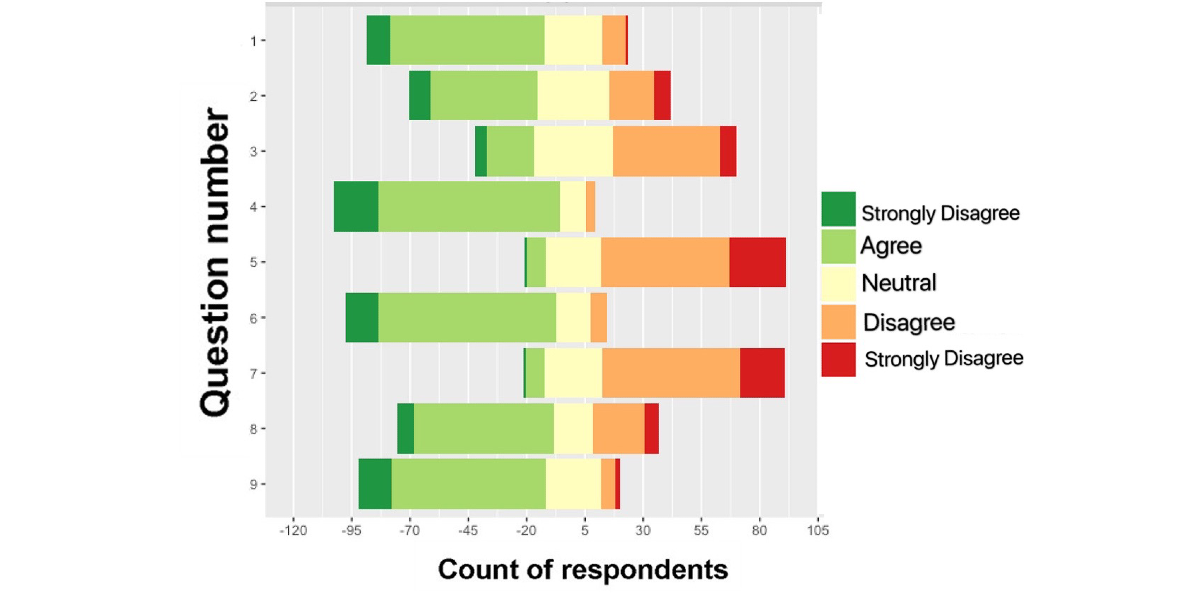
Diverging bar chart of responses of survey questions.

The dispersion of respondents’ opinions on each question was captured using a diverging bar chart (Figure 1). The counts for each question are presented on the x-axis, such that 0 is neutral, green indicates agreement, and orange and red indicated disagreement. Among the 9 questions, only 3 received greater than 50% of negative feedback. On the other hand, the nurses did not appear predominantly neutral on any one of the questions. Concerns over the EHR’s interference with direct patient care (Question 3) was the most reported issue among the 3 nursing EHR–related questions.

### Interquestion Associations

Kendall test was applied to assess the response resemblances among Questions 1, 3, 5, and 7 (Table 5). Nurses who reported higher satisfaction with the balanced workload between the EHR and direct patient care (Question 3) also tended to report higher satisfaction with EHR in general (Question 1; *P*<.001). On the other hand, those who were dissatisfied with the time spent in the EHR compared with direct patient care reported higher levels of stress (*P*<.001) and isolation (*P*=.009).

**Table 5:** Kendall test for question comparisons.

Comparison	Kendall test estimate	*P* value
Q1 versus Q3	0.3665	<.001
Q1 versus Q5	0.12	.15
Q1 versus Q7	0.0328	.69
Q3 versus Q5	0.337	<.001
Q3 versus Q7	0.1779	.03
Q5 versus Q7	0.216	.009

## Discussion

### Principal Findings

This study evaluated the relationship of EHR with satisfaction and well-being among ICU nurses across different units, age, clinical experience, gender, and EHR experience. We report that nurses were concerned regarding their EHR experience, in particular, the effect of EHR use on patient care. Nurses scored the highest satisfaction scores regarding their ability to manage the amount of assigned work. On the contrary, the proportion of time spent in the EHR compared with time on direct patient care was the lowest reported score, reflecting possible frustration.

### Generational Differences in Perceived Electronic Health Record Use

We report that age was negatively associated with the perception of the EHR affecting direct patient care, whereas older nurses reported that EHR time was not reasonable compared with patient care time. The correlation between age and perceived improved efficiency as a result of EHR adoption showed a significant negative association, as the younger nurses agreed that EHRs improved their efficiency compared with older nurses. The study results also expressed that the years of clinical experience had a significantly positive relationship with the ability to manage the amount of work, where nurses with more clinical experience reported a higher ability to manage work. This finding supports similar studies investigating a difference in perceived EHR satisfaction among physicians [16,23].

### Electronic Health Record Use and Nurse Well-Being

EHR satisfaction had significant associations with the overall well-being and feeling disconnected from work. ICU nurses’ survey responses showed a strong association between their EHR satisfaction and the frustration of being taken away from patient care, feeling worn out and weary, and the loss of passion or disconnection with work. Moreover, a strong resemblance was found between the frustration of spending too much time in the EHR with feeling worn out and weary and being more disconnected from work. Strong indications can show that EHR satisfaction can lead to feeling overworked and burned out.

To the authors’ knowledge, this is the first study to investigate the influence of EHR use on registered nurses’ well-being. Findings from this study are consistent with similar studies conducted on physicians and APRNs, wherein EHR use was found to contribute to professional well-being [15,17,21]. In addition, our findings validate previous studies suggesting that professional experience and age affect the perceived EHR experience [16].

### Findings From Previous Literature

Clinician well-being has been studied for decades [1,24]. However, contributing factors to well-being have been changing. In today’s world, an important contributor to well-being is the use of EHRs [25]. Many studies that reported on the relationship between EHR use and well-being focused on physicians [16,20,26-28]. However, significantly fewer studies explored the relationship between the well-being of nurses and EHR use [21,29]. Hoff et al [29] reported the lack of robust research designs to study well-being and high dissatisfaction among nurses.

A key study investigating the association between EHR use and APRN demonstrated a high correlation between EHR-related factors and well-being [21]. The study reports that 50% of the participants agreed or strongly agreed that the EHR added to their daily frustration, and 32.8% reported an insufficient amount of time for documentation. Our study validates this finding, such that 33% (37/113) of nurses reported dissatisfaction with the current EHR system. Moreover, 75% (85/113) of nurses did not believe the time spent in the EHR was reasonable compared with time spent in patient care. In addition, 50% (57/113) of nurses reported that the EHR did not improve their efficiency. Those reasons may explain the frustration with EHR systems and calls for more research to study ways to improve EHR satisfaction through improved interface design that meets the expectations of nurses.

### Limitations and Future Directions

This study was conducted as a single-site study, which may affect the generalizability of findings. Moreover, this study focused only on the perceptions of ICU nurses and assessed a single EHR system. Owing to the lack of a validated survey instrument to measure the impact of EHRs on the well-being of nurses, we designed a survey instrument on the basis of literature and domain experts’ input.

Future directions will include conducting a large-scale, multisite study to investigate if and how EHRs contribute to the well-being of nurses. This study provides baseline metrics for future usability research. Future studies will include conducting formal usability testing to complement findings from this study. Finally, more studies and comparisons are needed around this problem beyond critical care settings to build a more holistic picture.

### Conclusions

A significant part of a health care provider’s time is spent interacting with EHRs. We investigated the EHR satisfaction levels and the impact of EHRs on the well-being of nurses among ICU nurses at a tertiary medical center. Although nurses reported acceptable satisfaction scores with EHR use, deeper analysis suggests that EHR indirectly affects the well-being of nurses. We report a significant association between EHR satisfaction and nurses feeling burned out. More research is needed to improve the well-being of nurses through a better EHR interface design that meets the expectations of nurses.

## Appendix

Multimedia Appendix 1 Electronic health record (EHR)-burnout survey.

## Abbreviations

APRN: advanced practice registered nurse
EHR: electronic health record
ICU: intensive care unit
