# Supplementary material for: Understanding the Association Between Electronic Health Record Satisfaction and the Well-Being of Nurses: Survey Study
Source: JMIR Nurs. 2020 Jun 23;3(1):e13996. doi: 10.2196/13996 (PMC8279435; doi:10.2196/13996)
Supplement: Multimedia Appendix 1 [file nursing_v3i1e13996_app1.docx]

Appendix A

EHR-Burnout Survey

**Demographics**

Age: ______ Gender: ______ Unit: _________________

Highest Degree: (circle one) ADN BSN MSN DNP

Years of Experience Using the EHR: ________

Estimated # of Hours Weekly Using the EHR: _______

**Please answer the following question using the following 1-5 scale:**

1. Rate level of satisfaction with EHRs

Very Dissatisfied Dissatisfied Neutral Satisfied Very Satisfied

1 2 3 4 5

**Please answer the following questions using the following 1-5 scale:**

1. EHRs have improved my efficiency

Strongly Disagree Disagree Neutral Agree Strongly Agree

1 2 3 4 5

1. Amount of time I spend on EHR tasks related to direct patient care is reasonable.

Strongly Disagree Disagree Neutral Agree Strongly Agree

1 2 3 4 5

1. Usually, I can manage the amount of my work well.

Strongly Disagree Disagree Neutral Agree Strongly Agree

1 2 3 4 5

1. After my work, I usually feel worn out & weary.

Strongly Disagree Disagree Neutral Agree Strongly Agree

1 2 3 4 5

1. I can tolerate the pressure of my work very well.

Strongly Disagree Disagree Neutral Agree Strongly Agree

1 2 3 4 5

1. Over time, one can become disconnected from this type of work.

Strongly Disagree Disagree Neutral Agree Strongly Agree

1 2 3 4 5

1. Lately, I tend to think less at work and do my job almost mechanically.

Strongly Disagree Disagree Neutral Agree Strongly Agree

1 2 3 4 5

1. I always find new and interesting aspects in my work.

Strongly Disagree Disagree Neutral Agree Strongly Agree

1 2 3 4 5
